# Exploring experiences and impact of the COVID-19 pandemic on young racially minoritised people in the United Kingdom: A qualitative study

**DOI:** 10.1371/journal.pone.0266504

**Published:** 2022-05-04

**Authors:** Rochelle A. Burgess, Nancy Kanu, Tanya Matthews, Owen Mukotekwa, Amina Smith-Gul, Intisar Yusuf, Isabella Lamptey, Nyisha McCauley, Renae Wilson, Michael Pirisola, Malik Gul

**Affiliations:** 1 Institute for Global Health, University College London, London, United Kingdom; 2 Wandsworth Community Empowerment Network, London, United Kingdom; National Institutes of Health, UNITED STATES

## Abstract

Within high-income-countries, the COVID-19 pandemic has disproportionately impacted people from racially minoritised backgrounds. There has been significant research interrogating the disparate impact of the virus, and recently, interest in the long-term implications of the global crisis on young people’s mental health and wellbeing. However, less work explores the experiences of young people from racialised backgrounds as they navigate the pandemic, and the specific consequences this has for their mental health. Forty young people (age 16–25) from Black, mixed and other minority backgrounds and living in London, participated in consecutive focus group discussions over a two-month period, to explore the impact of the pandemic on their lives and emotional wellbeing. Thematic analysis identified seven thematic categories describing the impact of the pandemic, indicating: deepening of existing socioeconomic and emotional challenges; efforts to navigate racism and difference within the response; and survival strategies drawing on communal and individual resources. Young people also articulated visions for a future public health response which addressed gaps in current strategies. Findings point to the need to contextualize public health responses to the pandemic in line with the lived experiences of racialised young people. We specifically note the importance of long-term culturally and socio-politically relevant support interventions. Implications for policy and practice are discussed.

## 1.0 Introduction

People from historically minoritised communities, particularly those from groups who face exclusions linked to race (racialised), have borne the greatest epidemiological, economic, social and psychological consequences of the COVID-19 pandemic [1–3]. While the social and emotional wellbeing of young people is of particular concern [4, 5], available literature is rarely disaggregated by race or ethnicity. This limits our ability to fully understand differential and compounded impacts created by structural differences, which elevate the risk of adverse mental health outcomes for young people from racialised backgrounds. For example, studies have explored the impacts of the pandemic on the mental health and wellness of young people from high-income-countries, in addition to investigating young people’s perceptions of their government’s response to the pandemic. A Portuguese survey exploring experiences of COVID-19 among people aged 16 to 24, found that the stressors carried biological, psychological and social impacts [6]. A UK survey of 2,036 young people aged 13–25, identified that 80% of respondents reported a deterioration of their mental health statuses, and heightened feelings of anxiety and loneliness due to the COVID-19 pandemic [7]. Singh et al.’s [8] narrative review of studies exploring the mental health implications of COVID-19 and lockdowns on young people and adolescents suggested a series of factors leading to this vulnerability. These include: developmental age, educational status, pre-existing mental health conditions, proximity to economic deprivation, heightened challenges experienced by school-aged children and college-aged young people from lower-income backgrounds. In a rare study exploring disaggregated data, a survey exploring abuse and mental health across the UK during the pandemic found that the youngest participants (18–29) from racially minoritised backgrounds faced the most significant risks of experiencing abuse, self-harm and suicidal ideation [9].

Scholars point to how structural inequities have made people from racialised background more susceptible to the virus and its corresponding financial and social outcomes [1, 10–12]. For example, a US study explored the differences in racial and ethnic minorities’ health experiences throughout the COVID-19 pandemic, finding they were more likely to have lost a job due to the pandemic, missed housing payments, faced food insecurity, and report higher feelings of anxiety [13].

Institutionalised exclusion and marginalisation have created a more volatile environment for racially minoritised people to weather this pandemic, both within the UK and across other high-income-countries. For example, protests against racist policing in the UK and the brutal murder of George Floyd by a Minneapolis police officer led to widespread attention to oppressive and racist policing practices facing racially minoritised people globally. In the UK, acknowledgment of oppressive and racist practices in policing forced many to confront their own heightened vulnerability to a new global health threat and its connections to long standing racism and systemic oppression [14].

A growing body of evidence highlights the negative impact that racialised experiences of everyday life have on health outcomes for adults from racially minoritised backgrounds [15–17]. However, there is limited literature exploring the mental health experiences of young people from these communities and its connections to deepening precarity and mental health consequences. Our work contributes to this gap, through exploring the following research questions: *How have young people from racially minoritized communities been affected by the pandemic*? *How does this relate to their emotional wellbeing*, *and how do they cope with these new sets of challenges*? *And*, *importantly*, *how can programmes better support their needs*? Our work is shaped by a non-medicalised framing of mental distress, in order to maintain an emphasis on the downstream factors and symptoms that may lead to the development of poor mental health outcomes in the future [18].

## 2.0 Methods

### 2.1 Research design and setting

This study was approved by the University College London (UCL) research ethics committee (REC) [ID Project ID 16127/003]. We used qualitative research methods to explore the perspectives and experiences of young people aged 16–25 from racially minoritised backgrounds living in London, regarding COVID-19 and the initial lockdown period in the UK. A repeated focus group design was conducted over eight weeks to explore young people’s experiences, perceptions of the response, and coping strategies amidst the pandemic. The design emphasized a strength-based exploration, which counters a deficits-based approach by interrogating resilience and survival within communities in the face of socio-political stressors. Strength-based exploration has proven to be a valuable platform for supporting development of more meaningful health programs and health enabling environments [19–21].

The study was informed by co-design principles [22], working in partnership with Wandsworth Community Empowerment Network’s (WCEN) Black Minds Matter (BMM) collective; a London based youth activist organisation committed to advocating for and improving young racially minoritised people’s mental health and wellbeing. Peer facilitators [BMM members aged 20–30] contributed across the life of the project, informing the research design, collection and analysis of data as part of the research team. Methods and analysis are reported in accordance with the consolidated criteria for reporting qualitative research (COREQ) guidelines [23].

### 2.2 Participants and sampling

Forty participants between the ages of 16 to 25 were recruited to two cohorts, broken into three age groups (16–17; 18–20; 21–25). Participants were placed in groups with their age categories, which meant there were three groups per cohort. In line with UK NHS guidelines on research with young people, individuals aged 16 and up are able to consent for their participation independently of parental consent, and this approach was approved by our UCL REC. In cohort 1, all participants were purposively sampled from within the WCEN BMM collective. This was to ensure an existing level of trust between participants and researchers given the sensitive nature of the topics being explored. The value of peer-based approaches among marginalised groups is well known, particularly for its ability to establishing trust among groups labelled as ‘hard to reach’ [24]. The second cohort were identified through snowballing sampling methods, recruiting from additional youth organisations in London linked to WCEN. Peer facilitators were members with connections to these organisations. This allowed us to diversify our perspectives, while maintaining a common thread between groups participants. Demographic details are presented in Table 1.

**Table 1:** Participant’s demographics.

Characteristics		*N* (%)
	Total Participants	40 (100%)
**Race/Ethnicity**		
	Black African and or Black Caribbean	35 (87.5%)
	Mixed Ethnicity	2 (5%)
	South Asian	3 (7.5%)
**Sex**		
	Female	32 (80%)
	Male	8 (20%)
**Age**		
	16–17	10 (25%)
	18–20	20 (50%)
	21–25	10 (25%)
**Education**		
	College*	23 (57.5%)
	Undergraduate/ University	12 (30%)
	Postgraduate/ Masters	5 (12.5%)
***Postcodes within Greater London area***	2^nd^ decile	1 (2.5%)
	3^rd^ decile	8 (20%)
	4^th^ decile	12 (30%)
	5^th^ decile	5 (12.5%)
	6^th^ decile	10 (25%)
	9^th^ decile	1 (2.5%)
***Postcodes outside of Greater London area***	8^th^ decile	1 (2.5%)
	9^th^ decile	2 (5%)

* College in the UK meaning, the preliminary schooling that people may do in preparation for an Undergraduate degree at a University, and or where people can learn vocational skills

** Index of Multiple Deprivation (IMD) decile index is how relative deprivation is measured in England [25]. The British Ministry of Housing Communities & Local Government calculates IMD scores annually, within deciles ranging Lower-layer super output area (LSOA) or neighbourhood scores from 1, being most deprived, to 10, being least deprived. The IMD scores are the most current published data and are from 2019 [26]. For this research, the IMD scores presented were calculated by averaging all of the IMD scores from the first half of participants’ postcodes within the local authorities.

Recruitment was conducted by email and telephone discussions, and handled by the WCEN project manager, who was also available to answer any research queries. All participants were informed of the benefits and potential risks of participating in this study, given participant information sheets regarding the study, and signed informed consent forms electronically given remote data collection strategies in place due to the pandemic. Consent was re-affirmed verbally at the start of each discussion group.

### 2.3 Data collection

Focus groups were organised by age (see Table 2) to account for differences in life circumstances of adolescents and young adults. Facilitators worked in pairs to run each group; combining an academic or senior staff of WCEN member, with one of our peer facilitators. All groups were trained on the data collection protocol (topic guides, ethical protocols) by RAB. Sessions were facilitated using a topic guide, exploring participants’ experiences and perspectives of COVID-19 and government’s response, coping strategies, and planning for how policy and communication could be improved to better support young people from these backgrounds. The topic guides were drafted by RAB and revised following discussions with peer facilitators, given their proximity to the experiences being explored in the guides.

**Table 2:** Age demographic focus group section assignment by recruitment cohort.

		Age Group	Number of Participants Enrolled
Cohort 1	Group 1	21–25	5
	Group 2	18–20	5
	Group 3	16–17	5
	Group 4	16–17	5
Cohort 2	Group 5	18–20	4
	Group 6	18–20	5
	Group 7	18–20	6
	Group 8	21–25	5

Each group met four times, fortnightly over eight weeks. Facilitators reminded participants during each focus group meeting of their right to withdraw from the study. The focus group discussions were held on zoom on an institutional account, were audio recorded and lasted an average of 60 minutes. During each focus group discussion, facilitators were given a link to a password-protected, live Google document that allowed for anonymous editing. Google documents were available at the start of each session, but the amount they were used varied between groups. The prompt for the documents, was to use them if at anytime they felt uncomfortable sharing their perspectives or feelings verbally. Some groups used them quite prescriptively, with participants typing their answers to questions in the topic guides. Other groups used them sparingly, opting to discuss verbally responses to questions. These differences were determined by the natural flow of the groups, with facilitators following the lead of participants in the choices they made, rather than making suggestions in any direction of what was preferred mode of data collection.

### 2.4 Analysis

Each group (n = 8) met four times, resulting in 36 total focus group discussions (FGD). In this study we focus on 24 FGDs that explored topics most pertinent to our study questions (impact of the pandemic and desires for public mental health policy). Of these FGDs, 20 audio recordings were transcribed verbatim by NK. Four recordings were not clear enough for transcription purposes and google documents for these groups were used instead. All participants were anonymised at point of transcription. Microsoft excel was used to organize and manage data.

Data was analysed iteratively using of Braun and Clarke’s [27] thematic analysis (TA) method, following all six steps: (1) familiarisation with data; (2) generation of initial codes; (3) searching for themes; (4) reviewing the themes; (5) defining and naming themes; and lastly (6) producing the final report. A group analysis was coordinated to ensure the full involvement of young people throughout. Our analysis blended inductive/deductive orientations to the data. We wanted to remain anchored to the conceptual ideas of interest in our question, but not blind to the ways in which participants interpret and make sense of these concepts in their own lives. In reporting our analysis, we also had a desire to build deeper thematic arguments, pointing to processes or complex collection of actions within our coding process. We pulled together themes that related to each other into seven higher order themes ‘thematic categories’. This approach to labelling reflects our desire maintain the processes and notion of a higher order theme as used by Braun and Clarke in TA, but helps us to better differentiate the existence of a new ‘category’ of ideas within the data. We feel that approach is similar to the approach taken in thematic network analysis [28], which aims to brings together sub-themes and themes in ways that generate specific argumentative claims about what our analysis uncovered.

RAB and NK familiarised themselves with all of the focus group transcripts, analysing them by age cohorts to explore cross cutting themes. Each independently coded a set of FGDs (NK: 16–17, RAB: 21–25), then devised an initial structure of a coding framework to organise data driven codes using six categories loosely guided by the topic guides. Transcripts from the 18–20 cohort were distributed to peer facilitators and senior facilitators, who independently coded and identified data driven codes. These transcripts were selected as it was the largest sample and allowed us to ensure that each team member worked with a different piece of raw data. Peer researchers were given one week to read through their transcript, and were instructed to look for patterns in the text, and to label each pattern that they thought was important to the story of their transcript (theme generation). Where possible, peer-researchers were given transcripts for sessions facilitated by someone else. A team analysis meeting was held on zoom and consisted of two parts. During the first hour, RAB facilitated a discussion, oriented around a shared excel screen. Each peer-researcher shared the themes that they identified in their analysis, which was used to build a framework live by RAB. This created a bottom-up thematic framework. The second hour, looked at the similarities and differences between this framework, and the initial framework developed by RAB and NK. After refinement, an additional thematic category was created. Similarities and differences across age group categories were noted, (see coding frameworks in S1 File) but none of these were felt to be salient enough to shift the primary stories generated from the data by the team as a whole. Following the team analysis meeting, NK refined our proposed framework, by re-reading a selection of transcripts applying the final coding framework. No changes were made. The thematic framework was approved by the research team, after which a visual summary of our findings (thematic word cloud) was shared with participants via whatsapp, to give them an opportunity to validate the claims of the full analysis. [25–27, 29, 30]. Peer researchers also verified the initial written draft of the results section to ensure consensus about reporting of findings and perspectives on potential implications of the work. This approach further reduced barriers for peer researchers’ engagement in the breadth of the academic process [31, 32].

## 3.0 Results

Analysis produced seven thematic categories (higher order themes) which described the impact of and efforts to manage the pandemic by young people: (1) socioeconomic challenges: deepening current burdens and future instability, (2) emotional challenges: disruptions to life and fears of the future, (3) government’s response: too much too soon and lack of clarity, (4) navigating racism and difference during the pandemic response, (5) survival during crisis: finding the new you in this new situation, (6) community: transitions, successes, and spirit, and (7) visions for a future response.

### 3.1 Socioeconomic challenges: Current burdens and future instability

The socioeconomic challenges faced by young people were consistent across all age cohorts, specifically the experiences of financial insecurity. Despite just over 30% of participants residing in less deprived areas, this continuity of difficult economic experiences coincides with the distribution of poverty in London, where inequalities exist even within the same local geographical areas [33]. Parents had lost income, and young people had also lost work, increasing feelings of precarity. Concerns were intensified as government policies seemed to create new financial strain on their lives. For example the potential loss of access to subsidised travel for children under 18 in London was frequently discussed, with one participant stating:

It’s a lot. It’s like over a pound when I get on the bus, so I really put on like seven pounds on if I’m like going different- if I’m taking different busses. They still have the cap, I think. But it’s just stress. I can’t be bothered to get on the bus anymore because I have to pay. *(Focus Group 4*, *16-17-year-old)*

Many worried about how this would affect their ability to move around the city, which would have knock-on consequences for other issues, such as schooling or engaging in employment. Participants also expressed insecurity about the future, highlighting frustration regarding the lack of job opportunities available, and the impact this would have on their future, as many relied on the industries that were most disrupted by the pandemic:

Another thing is, um, I was looking for part-time jobs during the pandemic, which is really stupid, but I still wanted to try so like when they reopen. Hopefully I get a job or something like that. I can’t remember who said it, but one of the MPs—I’m not sure if he’s a MP, but they said that they would increase more jobs for young people, especially those under 18. And I trusted them and it’s not working. I don’t see any jobs for us. *(Group 4*, *16-17-year-old)*

### 3.2 Emotional challenges: Disruptions to life and fears of the future

In addition to distress from expected consequences, such as grief related to covid-related deaths among family, and missing friends and relationships, participants noted specific emotional challenges that they attributed to government management of the pandemic. These linked to new worries about school, stark changes to routines and uncertainty as the UK eased out of its first lockdown in July 2020.

Young people across all age groups struggled with deepening of existing or the emergence of new mental health difficulties as a result–most notably anxiety.

For younger participants specifically, unclear communication regarding school caused stress and anxiety, as noted by one young person: ‘*I feel like the closer we get to September the more anxious I get because I don’t know like I have no idea what’s gonna happen next year*.*’ (Group 4*, *16-17-year-old)*. Changes to routine established emotional challenges as highlighted by the anxiety felt by the absence of a routine, and the difficulties participants had in managing time is illustrated below:

….It’s like quite hard to adjust, especially since I was expecting school to like, come back the next month. I was like, it’s not that bad. I’ll just wait for school to come back. And it wasn’t ever—well it’s gonna come back, [but] it seemed like it wasn’t ever coming back because I was just like, I don’t know what to do…. I’m like, Okay, what do I do? *(Group 3*, *16-17-year-old)*

As the country began to ease out of the first lockdown in Summer 2020, participants worried that their social skills had been negatively impacted during isolation. They noted anxiety about reintegrating into the outside world, as lockdown restrictions were lifted, as illustrated in the following quote:

I feel like after corona and everything was announced and being locked in your house for so long… and then you go out- like for me personally… to be out for longer just would make me feel uncomfortable. Like I’d start feeling like I’d about to have an anxiety attack. Just because I don’t really know… you hear so much about what’s going on and how bad it is… It makes me worry more than I’m used to. *(Group 7*, *18-20-year-old)*

### 3.3 The government’s response: Lack of clarity, and too much too soon

Across all age groups, participants’ perceptions of the government’s response reflected feelings of frustration and disappointment. Beyond their distress at the loss of their ‘normal’ way of life, many felt that the government had misread the scale of the impact of the lockdown and virus on their lives. For example:

And I was just like these guys have really got their lives on hold. Thinking that one day things will eventually like go back to normal. And the government is just going to be like alright yeah that’s enough. Everyone go back to what you were doing before. And I was just realizing that too much has changed for that to happen. For everything to just go back. Like it’s gotten to a point where even if the virus like disappears off the face of the Earth. Things are not just going to return to normal. Like too much has changed. The economy has been impacted too much. People’s way of lives have been impacted. *(Group 5*, *18-20-year-old)*

Additionally participants expressed frustrations regarding the UK government’s handling of the pandemic and its impact on their emotional state. *‘But um I feel like*, *maybe specific to the UK*, *it wasn’t handled well*. *So the sense of panic was kind of amplified*.*’ (Focus Group 5*, *18-20-year-old)*. At the heart of this, was concern about poor government communication, disagreeing with the UK response, particularly lockdown policies:

But we’ve treated it as like, this unknown force of nature, that’s gonna wipe out half the population. And now, like, the approach—and that approach has just ruined a lot of people’s lives and set a lot of people back many years, months, whatever. *(Group 5*, *18-20-year-old)*

Young people also felt they were excluded from government COVID-related messaging, expressing frustration at being blamed for increases in cases, or systematic vulnerability to the virus:

I feel like, I don’t wanna say the government doesn’t care about us. But … I feel like young people are not a priority in the government’s mind right now. Because even like, when you go for information there’s nothing tailored for, for young people. *(Focus Group 5*, *18-20-year-old)*

This was compounded by their experiences of race related marginalisation within the response strategy. Participants across all age groups were acutely aware that people from Black and other minority groups were not only more vulnerable, but simultaneously being blamed for that vulnerability and the poorer outcomes within their communities. For example:

‘Well….they kind of blame the BAME community for not taking COVID seriously, when it’s hitting us the most.’ (Group 3, 16-17-year-old).

But still, I think there could have been a lot more clear, um, guidelines like even just in the past few months, when the government is encouraging a lot of young people and in particular, to go out and make use of the eat out, help out scheme. And they were encouraging that. And then within a matter of weeks, they’re now blaming young people, because the rates have been going up, so it’s such a contradiction, and I think they’re just looking for a scapegoat. And unfortunately, young people have become that scapegoat. Because they’re sending everyone back to school, sending everyone back to universities, but and then blaming them. So, I don’t know what message they’re trying to send out at the moment. But I don’t think it’s clear. *(Group 7*, *18-20-year-old)*

### 3.4 Navigating racism and difference during the pandemic response

As alluded to in the above theme, young people felt that there were racialized dynamics that deepened the psychological burden and impact of the pandemic in their lives. For example, young people described the dilemma that they faced as the Black Lives Matter movement surged in summer months; balancing a desire, and drive to participate in protests seeking to change their lives, alongside anxiety about their increased vulnerability to COVID-19:

I was just saying that I think like, that’s a dilemma a lot of Black people face because even I remember when I was going to the protest, my sister was like, I really, really want to go. My sister’s that has type one diabetes, and she was just like, ‘I’ll be risking my life’. And I was like, I don’t think it’s like, pro-Black to risk your life. I just think your Blackness is not defined by if you went to a protest, or if you even signed the petition, but like, like the fact that you’re living and just doing what you want to do, and having that autonomy. That’s what we all want to do. *(Group 1*, *21-25-year-old)*

Young people across age groups were sensitive to the mental health challenges they faced as a result of the pandemic. However, a desire to act on these issues often intersected with stigma related to mental health services held by older generations in their households. It is important to note that many participants consciously rejected stigma held by their elders, but noted the difficulties faced in accessing services more widely in their communities, as exemplified within this conversation:

Participant 2: Because they’re (community elders) thinking ’There’s nothing wrong with me. What? What are you talking about?’ But it’s like, they hear they hear certain words and obviously the stigma around it. So it’s gonna be a negative connotation. Like, it’s just 9 times out of 10 it’s just a prevention thing.

Participant 3: Yeah. And I think there’s the thing like, historically, the help hasn’t been there, like there has been no help to access. So it was sort of like, you have to be okay. Because you have no choice.

Participant 2: Yeah.

Participant 3: And that was instilled in people. So now even though the help is there, because we’re so used to um people, like, what not having our best interest at heart, we choose not to use it. *(Group 2*, *18-20-year-olds)*

Young people, particularly in older age groups, had a sense that their communities had been consistently left behind and overlooked by governments, and that the pandemic enabled much of the same to continue. As powerfully stated by one young person:

I’m fed up [with] thinking they’re *(the government)* going to help—they’re not going to help. They don’t care, we are not a priority to them, they have their own people, and they don’t care. Yeah. *(Group 1*, *21-25-year-old)*

In addition, many felt that the health communication strategies in place were ill-suited to their specific age, ethnic and cultural contexts. Among 16–17 age cohort in particular, it was felt that cultural and age representation was essential to ensure young people trusted the source of the information, which would contribute to their willingness to act on it.

Like, if you get an adult, the answer is not going to be able to relate to them. youngsters are like, I’m, I’m 17, you’re, you’re 27. How am I meant to relate to you? Like you’re giving me this information, but it’s not going to help me because look at you and look at me. *(Group 3*, *16-17-year-old)*

### 3.5 Survival during crisis: Finding the new you in this new situation

Participants described a range of new self-care strategies, defined broadly by all age groups as intentional practice that privileges and supports their physical and mental wellbeing.

Yeah I just wanted to stay healthy, you know. And I just wanted to be—just collect, look after myself in a sense. Self-care. (*Group 6*, *18-20-year-old)*

Um, I would just say focusing on myself more, okay. Just relaxing, making sure that I’m mentally okay. (*Group 6*, *18-20-year-old)*

Participants engaged in activities such as Yoga, listening to music, and spending a lot of time online. Some participants expressed their enjoyment of the increased free time they had as an unintentional outcome of the lockdown, stating:

I’m really, I’m really enjoying that. It’s just like sleeping when you want, waking up when you want. Just doing a couple of things from your bed, it’s just like I am really enjoying that time because I know I’m not going to get that again. And I think I’m just really enjoying taking the time for myself. *(Group 5*, *18-20-year-old)*

### 3.6 Community: Transitions, successes, and spirit

Participants highlighted the role of communities in responding to systemic neglect and establishing new modes of communication and engagement during and after the lockdown. Young people indicated how social media has become a site for community engagement and acknowledged their contributions to supporting other community members during this crisis.

Analysis drew attention to frustration felt towards the government’s disregard for the participants’ communities, noting that survival and community systems often needed to fill the gaps left by institutions, as illustrated below:

Um, I feel like if I left it to the government? No, definitely not. I know, I’m opening a youth club. So, I don’t, I’m not worried about them. But if I left it to them, no, no, they’re not worried about our problems. Our problems are our problems, and they need to be dealt with by us. In their eyes. *(Group 1*, *21-25-year-old)*

Participants noted the heightened importance and power of community in their lives during the pandemic. They highlighted expansion of existing support and developments of new relationships and scaffolding that had not previously existed, to respond to personal, financial and social emergencies faced by the community:

During the lockdown time and my neighbours had a church group, and they were giving out money. Well not giving out money. They were paying people’s rent fines, like things that people couldn’t afford bills, and stuff like that. They supported me a bit during lockdown. *(Group 1*, *21-25-year-old)*

Throughout periods of prolonged physical isolation, how young people gather and engage with their communities has evolved to meet the moment. Analysis demonstrated how young people turned to social platforms, as a new stie for community engagement during lockdown, as mentioned: *‘I watch a lot of YouTube so I like the content coming out I feel like because like everyone’s indoors um I feel like there’s more community base as well*.*’ (Group 5*, *18-20-year-old)*.

Analysis emphasised that young people were aware of their capacity to work individually and collectively for change. During the pandemic, young people’s awareness of their role within their communities were amplified, and they began to shift their actions, through checking in with others and joining online communities. and supporting neighbours or those viewed as vulnerable. For example, a participant who worked at a primary school shared their duty to ensure their stresses from the pandemic, and concurrent protests did not impact younger children they supported:

But being in that environment, I have to put their problems before mine because obviously if I come in with a negative energy and like they can see, I’m not really feeling like as positive as I should be as someone they’re meant to be like, looking to for help. I just didn’t want to be that person to like, bring them down even more. *(Group 2*, *18-20-year-old)*

The quote above stresses the duty shared by young people, as their concern for the difficulties wider community members have faced manifests as a responsibility to safeguard them, specifically from the amplified mental health difficulties throughout the COVID-19 crisis.

### 3.7 Visions for a future response

Our research also gave participants the opportunity to reflect on a new vision for future public health and public mental health responses to the pandemic. Perspectives varied between groups. For the youngest cohort, ages 16–17, Participants expressed appreciation for the platform provided by this research itself, and desires for peer-led COVID support groups, as they valued the opportunity to discuss their challenges with peers who have had similar experiences.

I said it in the previous sessions… a discussion group like this, it says, because it allows young people to kind of talk to each other about how they feel, instead of just holding it in. And just like, everyone can share their experience with each other. And then if you hear that someone’s experiences similar to yours, you won’t feel that lonely anymore. So, something like this, it doesn’t even have to be like a massive thing to just be with like, group for people. I just something like even in a place or an environment I like…we’ll just talk to each other about how you feel and how you’re coping with things. *(Group 3*, *16-17-year-old)*

This cohort also suggested that the government could improve communications about the pandemic with young people, by stressing a logic of care, and the importance of feeling like they connect with the speaker, as highlighted by the following quote:

Maybe it’s a young professional, basically giving the message out, because when it’s like an older white person giving me news, I don’t know, just. So maybe if there’s like a young person, you’d be able to trust them more kind of take more seriously. And it’s like, a person that seems like they genuinely care. Because I feel like a lot of the times people just say it because not as they don’t care. But it’s just like, if a young professional, just the person that seems like they genuinely care about you, and your well-being lots of people will be more interested in what they’re saying. It doesn’t even have to be a young professional, just a professional that seems to care about you. *(Group 3*, *16-17-year-old)*

The two older cohorts felt strongly that the government could improve its response by backing organizations and projects led locally by typically minoritised community members. In addition to arguing that young people should be involved in establishing communication strategies, participants within the oldest cohort highlighted the importance of enhancing ongoing work already being done within minoritised communities:

But maybe just amplify like, some of like the services that are already there… a lot of the mental health services and like services that are made by us like there’s so many people who are have got great youth projects, who’ve got like mentorship projects for Black children. And I feel like they’re not amplified enough. Like maybe in our community they are. But they’re not on the BBC, they’re not on like the NHS website. Like, I feel like the government needs to amplify people who are already doing the work. Because there’s a lot of people that are actually doing this work, and doing it last specifically for Black children. So, I feel like those voices definitely need to be amplified. *(Group 1*, *21-25-year-old)*

Lastly, all age groups agreed on the importance for compassion from systems they engage in. The youngest cohort (ages 16–17) asked for patience while navigating the pandemic and expectations for their school performance.

I think now that we’re going back to school, I think there should be more. I don’t know how to describe it, but like, a bit more understanding for young people because like for, for us, like I’ve made for myself, as soon as we go back to school, there’s like our mocks are coming up. And it’s understandable because we did miss out on doing our mocks, but I just feel like, there should be more understanding that young people are missing conditions at school. Like some people can’t replicate that working environment at home. So, to be more understanding, and like, maybe take off some of the pressure that is when young people, because we did miss out on a lot. And we had to go through, like a very rough time. *(Group 3*, *16-17-year-old)*

The oldest cohort expressed concern for the difficulties faced by young people, noting the potential impacts of these compounded struggles, and the need for action to mitigate this:

I think we are resilient people, but I just feel like these younger people, like, their resilience is unmatched, because they’re literally doing stuff that they shouldn’t have to do. Um, and also society’s just harder for them. Like, they’re gonna now pay to go to school, like, what is that? I mean, like, you can’t even have free travel. And it feels like so small, but it’s such a big thing. *(Group 1*, *21-25-year-old)*

## 4.0 Discussion

Through a series of focus group discussions with young people in London from racially minoritised backgrounds, our study highlights the intersections of existing and historical exclusions faced by their communities and how the pandemic’s worsening of those realities, created frustrations and distress.

Young people spoke specifically about loss of opportunities for income generation, and how this potentially shaped their futures, as the central concern in relation to socio-economic realities. Many participants in our study were from more deprived backgrounds, which have been among the hardest hit during the pandemic. Many continued to search for work, despite the disappearance of sectors where jobs normally existed. The newly developed anxieties and emotional distress linked to the loss of these opportunities poses a particular mental health concern for this group of young people, as recent evidence from Sweden indicates that employment precarity is a key social determinant shaping the development of mental health problems in previously healthy young people [34]. Given that many young black people face exclusion from appropriate mental health services in the UK [35], creating and cementing new pathways to mental health services is necessary as these types of pressures continue to mount in society.

Participants in this study also indicated the compounded difficulties of navigating the pandemic when negotiating their identity linked to their minoritised status. Many spoke of the ways in which their wellbeing was hindered by the push and pull of systems that simultaneously identified them as vulnerable, then blamed them for that vulnerability, and wider demands to be seen as protesting against the factors that drive their vulnerability. Consensus from work in other high-income settings like the UK, supports their experiences, noting the amplification of vulnerability faced by racialised minorities, particularly with regards to the mental health of Black people [36–38]. Our study’s findings contribute to this literature, emphasising the specific difficulties facing *young* Black people. This is significant, as these encounters are the starting point of a life-long exposure to the psychological pressures of paradoxical decisions that risk wellbeing on the one hand, in order to fight for access to rights, justice and change in their communities on the other. Recent work by Wakeel and Njoku [39] in the US, notes that these challenges occur across the life span, contributing to weathering of various aspects of health, including mental health. The compounding pressures of the pandemic and protests to end anti-Black racism among our participants forms a clear pathway to deepening mental health challenges of young people from racialised backgrounds, as the constant flow of information affirms Black people’s proximity to mortality, creating an additional burden that young Black people are forced to navigate [40, 41]. While literature from other high-income settings speaks of the different challenges and racialised experiences faced by young Black women and men [42], we did not note any gendered narratives of distress, or responses in our sample. It is worth noting, that given the predominance of young women in our sample, it is possible that the specific struggles of young men may have not become the core focus of discussions. With recent evidence highlighting that young black men face particular mental health crisis in this country [43], future studies may want to separate into groups by sex and or gender, to see what differences emerge in experiences, and responses to distress by young people.

However, despite challenges faced, there were consistent accounts of self-care across the age cohorts. Increasing such positive health behaviours has been supported by studies in other European contexts, such as in Portugal where Branquinho et al. [6] found that many young people enjoyed the free time they experienced due to lockdowns, as it created the time for personal development. Specifically, participants in our study described new habits such as meditation or exercising to keep physically and mentally well, which has been noted as beneficial to managing stressors caused by the crisis elsewhere in Europe [44–47]. As such, these strategies emerge as a starting point for expanding future public health strategies linked to the pandemic. However, we argue the need for these supports to be extended into the long term–as noted by many young people in our study, the impacts of the pandemic have changed the landscapes of their lives for the long term. The need to manage stressors will not be addressed by brief one-off interventions, and will likely require structural changes and the provision of economic resources to enable meaningful participation. This goes beyond free/low-cost services, but thinking about related incidental costs, such as travel, has been suggested as critical to securing good mental health in a post-pandemic UK [48].

### 4.1 Study implications and limitations

The pandemic has driven many researchers into online data collection, in response to social distancing guidelines. However, for participants in our study, connectivity issues and access to mobile data occasionally limited the ability of participants from the most minoritised backgrounds to participate in our study, which at times affected attendance. We tried to account for this in advance through our repeated focus group design, to increase opportunities for engagement. Furthermore, the online groups meant that audio recording from within laptops varied at times being inaudible or not recording at all (n = 4). The inclusion of google docs made for a useful backup option, particularly for groups where they were used in more depth, and highlights the value of triangulating methods for data collection within online qualitative studies.

The methodology used gave young people a chance to share and explore what they were feeling and experiencing, by acknowledging and reaffirming their pandemic experiences, and sharing strategies they used to navigate the crisis. This points to the value of research methods that are interested in paradigms of transformation–noting the importance of orientations and methods that acknowledge young people’s capacity for survival and positions them as experts in their own right [49]. As mentioned above, this work is potentially limited by the lack of a gendered focus on impacts and coping, and future studies may want to interrogate these points further. Finally, this project presents a snapshot in time of the perspectives of young people during the pandemic. However, many of the issues raised are anchored to structural difficulties that were illuminated, not created by, the pandemic, which maintains their relevance to the current political moment.

In Table 3, we summarise six areas of importance highlighted by young people, alongside recommendations stemming from the calls for action raised within discussions.

**Table 3:** From struggles to solutions–recommendations to support emotional wellbeing of young Black and minoritised young people in during and post-pandemic.

Thematic Category	Theme	Impact	Recommendations
1. **Socioeconomic challenges: current burdens and future instability**	• Insecurity about future	• *Interruption of school & concerns of school progressionFeeling lost and stuck* • *Uncertainty about employment*	• Maintain financial support for families beyond the pandemic • Protecting work/internship schemes for vulnerable young people from hard hit communities
2. **Emotional challenges: disruptions to life and fears of the future**	• Mental health conditions and emotional wellbeing	• *Impact of isolation on mental health* • *Stress from school* • *Stress from changes to routine* • *Stress and anxiety from the UK easing out of the first lockdown in July 2020* • *Concerns of social skills being negatively impacted from prolonged isolation*	• Online peer support networks connecting young people in regular conversations • Staged transitions back to the ‘real world’ as young people reengage with wider communities • Support for schools to manage increased anxiety among young people about their futures
3. **The government response: too much too soon and lack of clarity**	• Feeling excluded from government messaging and approach	• *Government’s approach to the pandemic caused a loss of ‘normal’ way of life* • *Government messaging was not tailored and inaccessible*	• Giving young people ownership over health messaging to ensure that it is targeted to their needs
4. **Navigating racism and difference during the pandemic response**	• Psychological burden of negotiating racial minoritised identity	• *Lockdown gave time to reflect on struggles within their communities* • *Importance of representation (e*.*g*. *similar age*, *race/ethnicity*, *socioeconomic background) when accessing support services and interpreting messaging*	• Including young people in design of racially sensitive communications around the pandemic
5. **Survival during crisis: finding the new you in this new situation**	• Finding joy within lockdown	• *Creative ways to find joy amidst crisis* • *Prioritisation of self-care practices* • *Adoption of new hobbies and habits*	• Reducing barriers to access self-help support for young people (i.e–free data packages for vulnerable households)
6. **Community transitions, successes and spirit**	• Community evolution from pandemic	• *Community filling gaps left by government failings*	• Funding and financial support for local community organisations responding to pandemic

Overall, participants communicated feelings of being overlooked and excluded from the government’s response to the virus. As messages and services are not generally tailored for young people, participants emphasised the need to develop bespoke messaging for young people to communicate similar information as that which was given to the adult population. Government and public health officials should cultivate a youth-friendly messaging stream to release critical information and changes to the rules, as a significant component of society felt excluded, forgotten, frustrated and anxious during initial stages of the pandemic. Consequently, visions for future public health responses reflected a desire for compassion from wider institutions in light of the difficulties that they experience pre- and post-pandemic. We suggest that systems can best show compassion for the pressures on young people’s lives, by responding not just to emotional distress, but also to the social and political drivers of uncertainty in their lives; protecting jobs and internship schemes, increasing financial resources to vulnerable households and reducing many economic barriers to participating in social life as we reopen society and communities. Given that recent months have witnessed the rolling back of supports to many vulnerable families despite the ongoing need for support, the calls made by young people in our study remain critical for UK policy planning.

## 5. Conclusion

Almost a year on from our project start date, the pandemic rages on. The disproportionate impacts on communities of colour remains across the U.K. [50]. Young people from minoritised backgrounds are a continued focus of social welfare interventions, often designed on their behalf. Within these efforts, we often fail to recognise their continued survival in the face of adversity, as a process that makes them experts in their own right.

Alongside young co-researchers, this study sought to understand how the pandemic affected young people from racialized backgrounds. Through a repeated measure focus group design, we explored how young people’s mental health and well-being were affected by the pandemic and related policy, highlighting barriers and coping strategies and solutions. While participants in the study expressed frustration with the government messages, which were consistently identified as unclear and unfairly targeted, participants also illuminated survival mechanisms and practised self-care to cope during the pandemic. Importantly, they had clear accounts of what needed to be done to make messaging and responses better suited to their needs; highlighting the importance of establishing online peer-support groups, and financial backing for local community-based and -led strategies targeting the COVID-19 pandemic and related emotional and social struggles. As evidence of impacts of racialised experiences impact the mental health of young people continues to emerge nationally [51], it is our hope that this work amplifies young people’s ongoing experiences of the pandemic and enables explicit responses from wider systems in the face of an ongoing crisis, for those hardest hit.

## Supporting information

S1 File Supplementary data tables–Thematic frameworks and sample codes/quotations. (DOCX)
