# Supplementary material for: Exploring experiences and impact of the COVID-19 pandemic on young racially minoritised people in the United Kingdom: A qualitative study
Source: PLoS One. 2022 May 4;17(5):e0266504. doi: 10.1371/journal.pone.0266504 (PMC9067664; doi:10.1371/journal.pone.0266504)
Supplement: S1 File — (DOCX) [file pone.0266504.s001.docx]

Supplementary data: Thematic frameworks Appendices

A1. Socioeconomic challenges thematic framework table

| Colour coding by age group | |
| --- | --- |
|  | Multiple Ages |
|  | 16-17 |
|  | 18-20 |
|  | 21-25 |

|  |  |  |
| --- | --- | --- |
| *Thematic Category:* | **Socioeconomic challenges** | |
| *Themes* | *sub-themes* | *Sample Codes* |
| Money problems | Loss of financial support (eg. bus passes); Economic issues caused by pandemic | It’s a lot. It's like over a pound when I get on the bus, so I really put on like seven pounds on if I'm like going different- if I'm taking different busses. They still have the cap, I think. But it's just stress. I can’t be bothered to get on the bus anymore because I have to pay. *(Focus Group 4, 16-17-year-old)* |
|  |  | I think children should be, I feel like children in poverty should be getting resources for free because it will help them for their future as well. *(Focus Group 4, 16-17-year-old)* |
|  |  |  |
| Insecurity about future | Finding work; Career prospects; Lack of general opportunities | Another thing is, um, I was looking for part-time jobs during the pandemic, which is really stupid, but I still wanted to try so like when they reopen. Hopefully I get a job or something like that. I can't remember who said it, but one of the MPs - I'm not sure if he's a MP, but they said that they would increase more jobs for young people, especially those under 18. And I trusted them and it's not working. I don't see any jobs for us. *(Focus Group 4, 16-17-year-old)* |

A2. Emotional challenges thematic framework table

| Colour coding by age group | |
| --- | --- |
|  | Multiple Ages |
|  | 16-17 |
|  | 18-20 |
|  | 21-25 |

|  |  |  |
| --- | --- | --- |
| *Thematic Category:* | **Emotional challenges in the face of crisis** | |
| *Themes* | *sub-themes* | *Sample Codes* |
| Mental health conditions | Depression diagnosis; Anxiety; Negative mental health heightened during isolation | You know what I mean? Also like I was gonna say, when things like this if that person's also feeling comfortable enough to actually want to speak about these things, because sometimes things like that happen. You just - not want to be by yourself but you end up pushing other people away. Not on purpose, but because that's the only way you really know how to express how you're feeling and you may not know how to say how you are feeling. Because there are no words for it. (*Focus Group 2, 18-20-year-old)* |
|  |  | Yeah, I'd say like, in terms of mental health, because I tend to see a lot of like those kinds of scientists saying that a lot of young people's mental health have been or will be affected by this, but they haven't really given a medium where like, that can be helped. *(Focus Group 4, 16-17-year-old)* |
|  |  | Um, one of my friends during this current period, well it wasn't because of COVID. But COVID didn't help because she was now isolated, but she did, like become suicidal again. And it's because she was by herself. And like, we, like, we were her support system. And we couldn't reach out because when we couldn't, like, go out, she couldn't come. What could we do at this point? *(Focus Group 7, 18-20-year-old)* |
|  |  | I was I was in a bad place back then you know. But I just- I was not trying to leave my room. And then Corona happened and then I was like, wow, don't really gonna end bro sound like why would I leave my room when I wasn’t leaving my room before. Might as well just stay in my room now. *(Focus Group 5, 18-20-year-old)* |
|  |  |  |
| Change to routines | Changes in sleep; Anxiety caused by lack of a routine; Struggles with time management, creates and deepens anxiety | Not that it's like anyone's responsibility to do that for me. I know that I should do that. It's like quite hard to adjust, especially since I was expecting school to like, come back the next month. I was like, it's not that bad. I'll just wait for school to come back. And it wasn't ever - well it's gonna come back. It was like it seemed like it wasn't ever coming back because I was just like, I don't know what to do. I'm so used to people telling me what to do when I have to do something myself and decide. I'm like, Okay, what do I do? *(Focus Group 3, 16-17-year-old)* |
|  |  | Um I mean, I know I’m not managing my time, but I’m not too bothered about it. Because there’s not a lot going on for me. But there is a thing that I need to do for uni that I’ve been like pushing back for months now, and I should probably start that. But its because of the way that my schedule has changed this last week. So, I’m going to bed at like stupid hours, and then I’m waking up late and staying in bed for another couple hours. By the time I’m like ready to do stuff, I’m just like, there’s not actually a lot of time left. So I should probably do something about that, but at the same time, I’m not in a rush for it*. (Focus Group 5, 18-20-year-old)* |
|  |  | I felt like very like, confused. I didn't know what was going on. There was like - I wasn't really doing anything. Usually, I'd be up the school and stuff but the days were just like, all mixed together. Like it wasn't really a pattern anymore. It was discombobulated*. (Focus Group 6, 18-20-year-old)* |
|  |  | My sleep schedule was inverted. So you would never catch me during the day ever. But perhaps at night, you might catch me darting through the corridors to go to the kitchen or something. *(Focus Group 5, 18-20-year-old)* |
|  |  |  |
| Difficulty managing long-term conditions during COVID | Difficulty accessing health care, further creating isolation and anxiety; Disability; Managing long term health conditions; Health challenges | And I don't mind, during lockdown, I got quite sick because I was meant to have appointments because I um I have like arthritis issues. So literally once lockdown started, they cancelled my appointments. And so that kind of left me at home. And because of how severe the pain was getting, I couldn't really do anything. *(Focus Group 7, 18-20-year-old)* |
|  |  | This entire time we've just been hearing while people are dying people are sick, because it spreads so easily this, this, this this this. Its like, nothing to be productive in, you know, reassuring that we'll be okay. It's more like, if you catch it like you're most likely gonna die. So just keep that in mind type of thing. And that's just not been helpful to anybody really, especially like people who have previous existing health conditions, because it's like, they felt that- a lot of them, especially like people that I know, just didn't go out. And that's even unhealthy for anybody's mind, regardless of if you're sick or not. *(Focus Group 7, 18-20-year-old)* |
| Stresses over school | Exam scandal; Having to self-teach; Instability and anxiety over future | And on the news with the sixth formers who got real marks, and lost, they lost things, like not being able to go to universities they wanted to go to. That's worrying me, because if next year, another lockdown happens, like the pandemic gets worse again. Then my grades, maybe will end up like their's and I don't know what. So I'm worried about my future right now. *(Focus Group 4, 16-17-year-old)* |
|  |  | No, so we didn't get given any zoom classes, which was really hard on us. So, what they did was they just gave us like PowerPoints where we have to go through. And even though they gave us the PowerPoints, it wasn't that informative. So, more that, they gave us all the work, like telling us, you're doing all the work, they weren't there to like, guide us or help us. *(Focus Group 3, 16-17-year-old)* |
|  |  | I feel like the closer we get to September the more anxious I get because I don't know like I have no idea what's gonna happen next year. *(Focus Group 4, 16-17-year-old)* |
|  |  | Usually at this time I would be worried about like exams and stuff, like I would be prepping for it. But it all just ended so suddenly. So it was like, like it was like they just threw me into the deep end. (*Focus Group 6, 18-20-year-old)* |
|  |  |  |
| Easing out of lockdown | Anxiety reintegrating back into the 'outside world'; Stress about communication skills being negatively impacted from isolation; Feelings of indifference towards adapting to this new way of life | My mum was a bit worried, a bit worried. Yeah, because - well all parents are worried to be honest. But she was worried, because she thinks like, if I go out a lot of times she's gonna see me in a sense where I might catch the virus or I don't know something might happen. You know? (*Focus Group 6, 18-20-year-old)* |
|  |  | I will say seeing friends made me happier. Being able to have a bit of a social life. (*Focus Group 6, 18-20-year-old)* |
|  |  | And everyone was in the same boat. Which was yeah - which was kind of like sad as well. Cause everyone wants just wants to just have a normal life. It won't happen. And we just kind of have to deal with new changes. (*Focus Group 6, 18-20-year-old)* |
|  |  | I did feel a little shy or a little bit anxious, but I think it's because I haven't been ready to meet people that much. I mean, I don't communicate with people generally, but I think because I haven't been going to Uni, in quite a while. And talking to people- seeing people every week. But I think my ways of communication have kind of diminished a little bit. *(Focus Group 1, 21-25-year-old)* |
|  |  | I feel like after corona and everything was announced and being locked in your house for so long. And then you go out- like for me personally, I just - to be out for longer just would make me feel uncomfortable. Like I'd start feeling like I'd about to have an anxiety attack. Just because I don't really know, what like – with corona, you hear so much about what's going on and how bad it is. And you know, always be careful. It makes me worry more than I'm used to. *(Focus Group 7, 18-20-year-old)* |

A3. Perception of government response thematic framework table

| Colour coding by age group | |
| --- | --- |
|  | Multiple Ages |
|  | 16-17 |
|  | 18-20 |
|  | 21-25 |
|  | |

|  |  | |
| --- | --- | --- |
| *Thematic Category:* | **Perceptions of government response** | |
| *Themes* | *sub-themes* | *Sample Codes* |
| Loss of ‘normal’ way of life | Quick return to normal placed people at risk; Dislike of the government's push to normal; Families forced to work in unsafe conditions | And I was just like these guys have really got their lives on hold. Thinking that one day things will eventually like go back to normal. And the government is just going to be like alright yeah that’s enough. Everyone go back to what you were doing before. And I was just realizing that too much has changed for that to happen. For everything to just go back. Like it’s gotten to a point where even if the virus like disappears off the face of the Earth. Things are not just going to return to normal. Like too much has changed. The economy has been impacted too much. People’s way of lives have been impacted. *(Focus Group 5, 18-20-year-old)* |
|  |  |  |
| Poor messaging | Lack of clarity; Poor communication; Lack of trust in government; Lack of justification for guidelines (e.g. masks, rule of 6) | Um, I don't know. It just, I feel like a broken record. Just clarity. Just state what we need to do. Yeah, and especially in the way they were talking about how BAME individuals that are more susceptible to catch, the virus or like there's a high death rate for BAME individuals by saying that immediately. You know? Speak to BAME people. ‘Oh, whoa, this is a bit more dangerous for us’- which would, again inspire us to obey the rules more often. So again, just being clear. *(Focus Group 3, 16-17-year-old)* |
|  |  | Everyone has no idea what's going on. People are scared. People are dying. So, the higher places like government, people aren’t sure as well. It makes it worse for everyone else. So, I understand where they’re coming from, but also they need to like set down rules, which will apply to you. Not just ’Oh, you want to stay five feet apart? Or just stay six feet apart?’ It doesn't really make any sense.” *(Focus Group 4, 16-17-year-old)* |
|  |  | Yeah. Exactly. There isn't as much transfer. And the government isn't, or hasn't been certain on how things are being transferred, or how to regulate it. And I just feel like things have been taken a bit out of proportion, personally. *(Focus Group 7, 18-20-year-old)* |
|  |  | But um I feel like, maybe specific to the UK, it wasn't handled well. So the sense of panic was kind of amplified. *(Focus Group 5, 18-20-year-old)* |
|  |  | But we've treated it as like, this unknown force of nature, that's gonna wipe out half the population. And now, like, the approach - and that approach has just ruined a lot of people's lives and set a lot of people back many years, months, whatever. *(Focus Group 5, 18-20-year-old)* |
|  |  |  |
| Excluded from messaging | Feeling disconnected from traditional media sources; Feeling forgotten and overlooked; BAME people not being treated as a priority | I'm not really sure about Instagram so maybe that's a better way of showing it. Yeah, I think what's interesting too, I'd love to hear everybody else's opinion and perspective, but it seems like a lot of younger people don't really watch television like that. Like we stream and get our media elsewhere, but we don't really watch more traditional television. That's something like our parents would do. *(Focus Group 5, 18-20-year-old)* |
|  |  | So basically, the news that we're getting, but even different newspapers is like from the perspective and like from the view of that one person, which happens to be a wealthy white man. So, a lot of the times the news that we receive is not we can relate to, because we can't relate to that one. Wealthy white man, the way he sees things he sees, like, poor people as like an inconvenience. 'Why do we have to help them? They should work hard for it.' Whereas from our perspective, we're more understanding, but then we also can't relate to him because we could be like 'Oh, he has all this money, why can't he help us?'. *(Focus Group 3, 16-17-year-old)* |
|  |  | I feel like, I feel like, I don't wanna say the government doesn’t care about us. But I feel like they're, more focused on - I feel like young people are not a priority in the government's mind right now. Because even like, when you go for information there's nothing tailored for, for young people. *(Focus Group 5, 18-20-year-old)* |
|  |  | I was saying this to my mum as well, that they (*government officials)* weren’t even referring to us when they were saying young people on the TV. They were referring to, like, people aged like 20 to 40ish. And they kind of excluded us in their, like plans and explanations, there hasn't really been much. I don't know, room to explain anything for us. *(Focus Group 4, 16-17-year-old)* |
|  |  |  |
| Ending language of blame and shame | Frustration as government officials blamed young people for increases in cases; Feelings that young people were a scapegoat for second wave | But still, I think there could have been a lot more clear, um, guidelines like even just in the past few months, when the government is encouraging a lot of young people and in particular, to go out and make use of the eat out, help out scheme. And they were encouraging that. And then within a matter of weeks, they're now blaming young people, and because the rates have been going up, so it's such a contradiction, and I think they're just looking for a scapegoat. And unfortunately, young people have become that scapegoat. Because they're sending everyone back to school, sending everyone back to universities, but and then blaming them. So, I don't know what message they're trying to send out at the moment. But I don't think it's clear. *(Focus Group 7, 18-20-year-old)* |
|  |  | Exactly. And also, just going off that point, though, the way that they have kind of advertised this in a way like one of the government's like slogans is ‘protect granny’ or something like, yeah, it's kind of, it's kind of like saying, oh, young people are the reckless ones of our society, the ones that are basically ruining, like any sort of progression. Ruining any sort of like returning to normality. When, in actual fact, if you look at it, young people have been trying their best throughout all of it. But it's the government and like the way that everything is set up. It's not designed to protect young people, but they're designed to blame young people. *(Group 7, 18-20-year-old)* |
|  |  | Well or when they kind of blame the BAME community for not taking COVID seriously, when it's hitting us the most. (Group 3, *16-17- year -old)* |

A4. Survival during crisis – findings the new you in this new situation thematic framework table

| Colour coding by age group | |
| --- | --- |
|  | Multiple Ages |
|  | 16-17 |
|  | 18-20 |
|  | 21-25 |

|  |  |  |
| --- | --- | --- |
| *Thematic Category:* | **Survival during crisis - finding the new you in this new situation** | |
| *Themes* | *Sub-themes* | *Sample Codes* |
| Self-care strategies | Meditating; Avoiding social media during these times; Finding enjoyment during lockdown | I'd say for me, I haven't been on social media as weird as that sounds, like I've kind of tried to stay away from it. Because like, a lot of the time when I went on social media there was just like loads of negative things. *(Focus Group 2, 18-20-year-old)* |
|  |  | I'm really, I'm really enjoying that. It’s just like sleeping when you want, waking up when you want. Just doing a couple of things from your bed, it's just like I am really enjoying that time because I know I’m not going to get that again. And I think I’m just really enjoying taking the time for myself. *(Focus Group 5, 18-20-year-old)* |
|  |  | I would walk more, um eat healthier as well. To stay safer to be honest. Healthier. (*Focus Group 6, 18-20-year-old)* |
|  |  | Yeah I just wanted to stay healthy, you know. And I just wanted to be - just collect, look after myself in a sense. Self-care. (*Focus Group 6, 18-20-year-old)* |
|  |  | Yeah, um, I would just say exercising. For me personally, just for the stress. (*Focus Group 6, 18-20-year-old)* |
|  |  | Um, I would just say focusing on myself more, okay. Just relaxing, making sure that I'm mentally okay. (*Focus Group 6, 18-20-year-old)* |
|  |  |  |
| Creation | Creating new systems and activities of support; New ways to connect with people; New habits and routines; Starting new businesses | I'd say like currently, I'm a bit more optimistic compared to like, the middle of lockdown. I was like, the middle of lockdown hit me the hardest cause I was very unmotivated. I don't know if that's a word de motivated. Yeah, I didn't have any motivation at all to do anything. I just didn't see the point. And it was like just even simple tasks. I couldn't do it. Just like I just struggled to get out of bed. I just stayed in bed and listen to music and slept all day. So, I'm kind of getting out of that and getting up more recently, I started cooking a lot more because it's like a distraction at least have something to do. So, I realized I'm actually not that bad. I should have done it more often. *(Focus Group 3, 16-17-year-old)* |
|  |  | For some reason, when I spend the whole day doing one thing. When I can focus on one thing, I become extremely productive. So I kinda just make a schedule around that and assigned various things to to different days. So that I can come out of lockdown slash quarantine feeling like I didn’t they just waste months of my life. *(Focus Group 5, 18-20-year-old)* |
|  |  | So I started a eyelash company and a lipgloss company as well. Just like a cosmetic company. *(Focus Group 7, 18-20-year-old)* |
|  |  | Yeah, I got into a bit of art, just more basic drawings or more basic, like, for example, like sunsets. (*Focus Group 6, 18-20-year-old)* |
|  |  | So I'm like, there is no point in procrastinating because you could have gotten this done a week ago. You know? Let me just do it now. So, self disciplining myself. I think I've gotten better at that. Discipling myself. (*Focus Group 6, 18-20-year-old)* |
|  |  |  |
| Social support systems | Increased reliance and engagement with friends and family; Supporting friends and family who need you | I'm in managing my time. But maybe, I think maybe because I live with my parents that obviously, they're like, your parents like, helping you manage your time. Yeah. I just yeah. Other than that, if I was living by myself, I don't think l would be managing my time. *(Focus Group 5, 18-20-year-old)* |
|  |  | But now I get to see my friends. Even if it's just a come to the station and meet. You know? Because we kind of live close. (*Focus Group 6, 18-20-year-old)* |
|  |  |  |
| Agency | Belief that life will pass by, waiting on the government so people should take control of their own lives and maximize the situation to their benefit; Finding optimism in the current situation | I would say there is joy in the fact that things haven’t just crumbled to pieces. *(Focus Group 5, 18-20-year-old)* |
|  |  | Before I was procrastinating, saying I'll do it later, or later, and then another time. Okay, then next week Monday I'm gonna start. But now is the thing where I've got nothing else to do. I might as well do it, you know? So yeah, so as I said, I enjoy doing what I do now because I've kind of developed from from it anyway. (*Focus Group 6, 18-20-year-old)* |
|  |  | There’s a lot of people who aren’t used to making their own decisions and having their own agency when it comes to taking control of their life. You know they just pass through the system, go to uni, get a job, pay their taxes et cetera. But now you’re in a position where if you just sit down and wait for the government to tell you, you can go out now life will just pass you by. *(Focus Group 5, 18-20-year-old)* |
|  |  | Even though COVID’s been really bad, I would honestly say, not an advantage, but like most of like, the exams I had to do, were at home. So it meant that some of the exams I had to do were open book. So that is the advantage. But also for me personally, since everything is slowed down, it's given me more time to like develop myself. *(Focus Group 5, 18-20-year-old)* |

A5. Navigating racism and difference coding during the pandemic response thematic framework table

Colour coding by age group

|  | Multiple Ages |
| --- | --- |
|  | 16-17 |
|  | 18-20 |
|  | 21-25 |

|  |  |  |
| --- | --- | --- |
| *Thematic Category:* | **Navigating racism and difference during the pandemic response** | |
| *Themes* | *Sub-themes* | *Sample Codes* |
| Psychological burden | Anger; Psychological separation for survival; Negotiating Black identity or other minoritised identity | Especially it's like they're saying we're not allowed to go out and then that you want to be productive, and go to the protests, but then you've got to mind COVID-19 that's still out there. Now, and you're just trying to think of what's next. *(Focus Group 2, 18-20-year-old)* |
|  |  | And that was instilled in people. So now even though the help is there, because we're so used to um people, like, what not having our best interest at heart, we choose not to use it. In some of the same time, its like with the police. Historically, like Black communities haven't had a good relationship with the police. And even though now more lack people and stuff are trying to join the police to make it better, there's still a lot of stigma. Like if you say, 'oh im going to call the police' in the ghetto, people are going to look at you sideways. *(Focus Group 2, 18-20-year-old)* |
|  |  | I was just saying that, I think like, that's a dilemma a lot of Black people face because even I remember when I was going to the protest, my sister was like, I really, really want to go. My sister's that has type one diabetes, and she was just like, ‘I'll be risking my life’. And I was like, I don't think it's like, pro-Black to risk your life. I just think your Blackness is not defined by if you went to a protest, or if you even signed the petition, but like, like the fact that you're living and just doing what you want to do, and having that autonomy. That's what we all want to do. *(Focus Group 1, 21-25-year-old)* |
|  |  | So yeah. And even though there's a pandemic, I still feel like, it's good still, because racism is a killer. People get killed because the color of the skin, and it's not a choice. *(Focus Group 4, 16-17-year-old)* |
|  |  | It don't matter. Don't let them *(the government*) have power, do it for yourself, I'm fed up are thinking they're going to help - they're not going to help. They don't care, we are not a priority to them, they have their own people, and they don't care. *(Focus Group 1, 21-25-year-old)* |
|  |  |  |
| Importance of representation | Need to see people who look like you (eg. Similar age, race, socioeconomic background); People who understand your experience; Challenging stigma in BAME communities regarding seeking mental health support | I don't know if this is more of a personal thing, but sort of normalizing that as well. normalizing asking for help going out to like actively seek help when you feel you need it. Because especially in our community, that is not a normal thing. *(Focus Group 2, 18-20-year-old)* |
|  |  | But anyways, one big news company to kind of like that we can relate to that we can share our news, our truth, because we can only relate to ourselves, we can't expect that one white male that has like pressed pushing out his agenda into all these news companies, like newspapers, we can't expect him to relate to us because he's never going to relate to us. *(Focus Group 3, 16-17-year-old)* |
|  |  | Like, if you get an adult, the answer is not going to be able to relate to them. youngsters are like, I'm, I'm 17, you're, you're 27. How am I meant to relate to you? Like you're giving me this information, but it's not going to help me because look at you and look at me. *(Focus Group 3, 16-17-year-old)* |
|  |  | P2: Because they're *(community elders)* thinking 'There's nothing wrong with me. What? What are you talking about?' But it's like, they hear they hear certain words and obviously the stigma around it. So it's gonna be a negative connotation. Like, it's just 9 times out of 10 its just a prevention thing.  P3: Yeah. And I think there's the thing like, historically, the help hasn't been there, like there has been no help to access. So it was sort of like, you have to be okay. Because you have no choice.  P2: Yeah.  P3: And that was instilled in people. So now even though the help is there, because we're so used to um people, like, what not having our best interest at heart, we choose not to use it. *(Focus Group 2, 18-20-year-olds)* |

A6. Community: transitions, successes and spirit thematic framework table

| Colour coding by age group | |
| --- | --- |
|  | Multiple Ages |
|  | 16-17 |
|  | 18-20 |
|  | 21-25 |

|  |  |  |
| --- | --- | --- |
| *Thematic category:* | **Community: transitions, successes and spirit** | |
| *Themes* | *Sub-themes* | *Sample Codes* |
| Community systems at work | Community to fill institutional gaps and failures; Need for youth clubs | Um, I feel like if I left it to the government? No, definitely not. I know, I'm opening a youth club. So, I don't, I'm not worried about them. But if I left it to them, no, no, they're not worried about our problems. Our problems are our problems, and they need to be dealt with by us. In their eyes*. (Focus Group 1, 21-25-year-old)* |
|  |  | During the lockdown time and my neighbours had a church group, and they were giving out money. Well not giving out money. They were paying people's rent fines, like things that people couldn't afford bills, and stuff like that. They supported me a bit during lockdown. I didn't know that they were available via like social media and stuff. But they were, like more on my doorstep as my neighbours. *(Focus Group 1, 21-25-year-old)* |
| Community spirit evolution | Evolutions of ways to communicate with people; Changes in the meaning of community engagement | I'd say things like this, because a lot of the times like we’re just to young people out of many, but we have all these opinions. So, I'm pretty sure there are other young people that keep their opinions to themselves. So, I think by talking about these things, because a lot of the times, we just assume that someone's doing okay during this like lockdown, but then they could be having a hard time as well, if we was all open about it and spoke about our experiences to one another. *(Focus Group 3, 16-17-year-old)* |
|  |  | As I said you don’t need to parade around doing up superman or whatever. You just need to pay attention and just open your eyes and think. If there’s a situation where you could help are you going to go ‘Ah I’m too shy’ or ‘What if they tell me to get out of their face’ or something. Just uh be a bit more aware and look to see if there’s any way you can help. *(Focus Group 5, 18-20-year-old)* |
|  |  |  |
| Social media as site of community | Using social media to engage with community; social media as a safe space when among shared people | I watch a lot of YouTube so I like the content coming out I feel like because like everyone’s indoors um I feel like there’s more community base as well. *(Focus Group 5, 18-20-year-old)* |
|  |  |  |
| Responsibility to wider community | Deep concern for others beyond family; concern for others overrides lack of trust in other systems | And I feel like I've also learned to not put my feelings aside but at the same time, I put my feelings aside obviously for the other children because I obviously have my own problems going on. But being in that environment, I have to put their problems before mine because obviously if I come in with a negative energy and like they can see, I'm not really feeling like as positive as I should be as someone they're meant to be like, looking to for help. I just didn't want to be that person to like, bring them down even more. *(Focus Group 2, 18-20-year-old)* |

A7. Visions for a future response thematic framework table

| Colour coding by age group | |
| --- | --- |
|  | Multiple Ages |
|  | 16-17 |
|  | 18-20 |
|  | 21-25 |

|  |  |  |
| --- | --- | --- |
| *Thematic Category:* | **Visions for a future response** | |
| *Themes* | *sub-themes* | *Sample Codes* |
| Giving young people ownership | Government communication strategies will improve through giving ownership to young people | So if we kind of remove the idea that young people are useless, they don't do anything for our society, because a lot of the times older people just say that this generation is messed up, or this generation is like useless, or this generation, they just kind of put us down a lot. So we just end up seeing ourselves as just like, we're not helpful to society, there's no point in getting involved in anything. So, if we can just present ourselves and present young people as helpful that we need you like young people. ‘You're useful young people. We care about you, young people. You're special.’ Which is anything to be able to promote ourselves, and each other is just useful, we're not useless. *(Focus Group 3, 16-17-year-old)* |
|  |  |  |
| Peer support groups | Online platforms for young people to share their experiences with peers of COVID/lockdown | And like in a very healthy environment, where like, there was no judgment, no mockery or anything like that. I feel like that is something young people could do, because as well as helping themselves by like getting it off their chest, they're helping others because some people need to hear what others are going through to kind of like, think about what they're going through at the same time. So maybe more discussions like this will opportunities to have more discussions like this? And not only about just this lockdown, just other things in general. But other issues in general, because I don't think it should only stop with how we cope with Coronavirus. Like other issues like that young people face like maybe poverty or like racism, colourism or like just loads of issues that we all have, we should just have Black in a community where we can have these open discussions about it. And it's like, there's no right or wrong answer to anything that we're saying. Because it's your own perspective. It's your own experience. *(Focus Group 3, 16-17-year-old)* |
|  |  | I said it in the previous sessions… a discussion group like this, it says, because it allows young people to kind of talk to each other about how they feel, instead of just holding it in. And just like, everyone can share their experience with each other. And then if you hear that someone's experiences similar to yours, you won't feel that lonely anymore. So, something like this, it doesn't even have to be like a massive thing to just be with like, group for people. I just something like even in a place or an environment I like…we'll just talk to each other about how you feel and how you're coping with things*. (Focus Group 3, 16-17-year-old)* |
|  |  |  |
| Compassion and understanding from systems | Need for patience from institutions regarding the difficulties that young people in school have endured;  Recognition and understandings from systems about what people endure | I think it would, it would make young people feel like they're more supported in a way and that, like, we're also worried about you as well. It's just kind of like, I think because our family just wanted to make sure everyone's safe at home, but then they don't really realize the effects that is lockdown has on young people, and how young people might struggle to cope with it. So, it was just like grouping young people with every other group and within the society, and it's just not really fair to do that. Because I feel that young people have really struggled, not the most, but struggled a lot with the whole lockdown. Because we are missing school, we can't see our friends, it’s really stressful. And we're seeing a lot of things on social media, because young people spend a lot of time on social media, a lot of things have been going on. We're exposed to so much and it's like there wasn't it didn't seem there could have been it didn't feel that there was much support. *(Focus Group 3, 16-17-year-old)* |
|  |  | I think we are resilient people, but I just feel like these younger people, like, their resilience is unmatched, because they're literally doing stuff that they shouldn't have to do. Um, and also society's just harder for them. Like, they're gonna now pay to go to school, like, what is that? I mean, like, you can't even have free travel. And it feels like so small, but it's such a big thing. *(Focus Group 1, 21-25-year-old)* |
|  |  | I think now that we're going back to school, I think there should be more. I don't know how to describe it, but like, a bit more understanding for young people because like for, for us, like I've made for myself, as soon as we go back to school, there's like our mocks are coming up. And it's understandable because we did miss out on doing our mocks, but I just feel like, there should be more understanding that young people are missing conditions at school. Like some people can't replicate that working environment at home. So, to be more understanding, and like, maybe take off some of the pressure that is when young people, because we did miss out on a lot. And we had to go through, like a very rough time. *(Focus Group 3, 16-17-year-old)* |
|  |  |  |
| Improving government response by building in BAME contributions | Publishing accessible messages tailored to young people; Utilizing youth advisors to interpret COVID-related messaging for young people | But maybe just amplify like, some of like the services that are already there are a lot of the mental health services and like services that are made by us like there's so many people who are have got great youth projects, who've got like mentorship projects for Black children. And I feel like they're not amplified enough. Like maybe in our community they are. But they're not unlike BBC, they're not on like the NHS website. Like, I feel like the government needs to amplify people who are already doing the work. Because there's a lot of people that are actually doing this work, and doing it last specifically for Black children. So, I feel like those voices definitely need to be amplified. *(Focus Group 1, 21-25-year-old)* |
|  |  |  |
| Improved communication through connection | Successful communication linked to a logic/context of care; Fostering connection through those that are similar to you (eg. Similar age, race, socioeconomic background) | Maybe it's a young professional, basically giving the message out, because when it's like an older white person giving me news, I don't know, just. So maybe if there's like a young person, you'd be able to trust them more kind of take more seriously. And it's like, a person that seems like they genuinely care. Because I feel like a lot of the times people just say it because not as they don't care. But it's just like, if a young professional, just the person that seems like they genuinely care about you, and your well-being lots of people will be more interested in what they're saying. It doesn't even have to be a young professional, just a professional that seems to care about you. *(Focus Group 3, 16-17-year-old)* |
|  |  | I'd say someone who's able to communicate with them. Cause, say if they want to go with someone who doesn't know anything about what they're going through, it will be harder to talk to them. Because, for example, as we said, if I if one of us were to go to like a white person and try and tell them what we're going through, like at family, at home, they wouldn't be able to understand anything. Even like with expectations. I'm quite I'm pretty sure it's common with all like being community and families. They have high expectations. I won't say when we go to like no offense for like a white family and we told them all. This is what they'll be like. Not even that hard, like they wouldn't understand where we're coming from. *(Focus Group 3, 16-17-year-old)* |
